# A Walnut-Enriched Diet Affects Gut Microbiome in Healthy Caucasian Subjects: A Randomized, Controlled Trial

**DOI:** 10.3390/nu10020244

**Published:** 2018-02-22

**Authors:** Charlotte Bamberger, Andreas Rossmeier, Katharina Lechner, Liya Wu, Elisa Waldmann, Sandra Fischer, Renée G. Stark, Julia Altenhofer, Kerstin Henze, Klaus G. Parhofer

**Affiliations:** 1 Department of Internal Medicine 4, Ludwig-Maximilians University Munich, Marchioninistraße 15, 81377 Munich, Germany; charlotte.bamberger@med.uni-muenchen.de (C.B.); andreas.rossmeier@med.uni-muenchen.de (A.R.); katharina.lechner@mri.tum.de (K.L.); wuliya@pennmedicine.upenn.edu (L.W.); elisa.waldmann@med.uni-muenchen.de (E.W.); julia.altenhofer@med.uni-muenchen.de (J.A.); kerstin.henze@med.uni-muenchen.de (K.H.); 2 ZIEL—Institute for Food & Health Core Facility, Technical University Munich, Weihenstephaner Berg 3, 85354 Freising, Germany; sandra.fischer@tum.de; 3 Helmholtz-Zentrum Munich, Institute for Health Economics and Healthcare Management, 85764 Neuherberg, Germany; r.stark@helmholtz-muenchen.de

**Keywords:** walnuts, nuts, diet, gut microbiome, lipids, cholesterol, prebiotic, probiotic, butyric acid

## Abstract

Regular walnut consumption is associated with better health. We have previously shown that eight weeks of walnut consumption (43 g/day) significantly improves lipids in healthy subjects. In the same study, gut microbiome was evaluated. We included 194 healthy subjects (134 females, 63 ± 7 years, BMI 25.1 ± 4.0 kg/m^2^) in a randomized, controlled, prospective, cross-over study. Following a nut-free run-in period, subjects were randomized to two diet phases (eight weeks each); 96 subjects first followed a walnut-enriched diet (43 g/day) and then switched to a nut-free diet, while 98 subjects followed the diets in reverse order. While consuming the walnut-enriched diet, subjects were advised to either reduce fat or carbohydrates or both to account for the additional calories. Fecal samples were collected from 135 subjects at the end of the walnut-diet and the control-diet period for microbiome analyses. The 16S rRNA gene sequencing data was clustered with a 97% similarity into Operational Taxonomic Units (OTUs). UniFrac distances were used to determine diversity between groups. Differential abundance was evaluated using the Kruskal–Wallis rank sum test. All analyses were performed using Rhea. Generalized UniFrac distance shows that walnut consumption significantly affects microbiome composition and diversity. Multidimensional scaling (metric and non-metric) indicates dissimilarities of approximately 5% between walnut and control (*p* = 0.02). The abundance of *Ruminococcaceae* and *Bifidobacteria* increased significantly (*p* < 0.02) while *Clostridium* sp. cluster XIVa species (*Blautia*; *Anaerostipes*) decreased significantly (*p* < 0.05) during walnut consumption. The effect of walnut consumption on the microbiome only marginally depended on whether subjects replaced fat, carbohydrates or both while on walnuts. Daily intake of 43 g walnuts over eight weeks significantly affects the gut microbiome by enhancing probiotic- and butyric acid-producing species in healthy individuals. Further evaluation is required to establish whether these changes are preserved during longer walnut consumption and how these are linked to the observed changes in lipid metabolism.

## 1. Introduction

The human gut microbiome encompasses approximately 1014 resident microorganisms, mainly consisting of bacteria, and corresponds to 1000 distinct species with a collective genome containing at least 100 times as many genes as the human genome [1]. The establishment of high-throughput sequencing allows the metagenome to be studied for broad analyses of intestinal microbiota composition [2]. These microbial communities contribute to host health through various functions including probiotic properties, biosynthesis of vitamins and essential amino acids, as well as production of metabolic byproducts from indigestible dietary constituents. Butyrate, a short chain fatty acid which is produced by bacterial fermentation of non-digestible carbohydrates in the colon, acts as a major energy source for intestinal epithelial cells, enhances intestinal epithelial barrier function and modulates immune function [3,4].

The fact that there is considerable variation in the constituents of the gut microbiota among apparently healthy individuals strengthened the hypothesis that there is a clear link between health, disease and diversity of the human gut microbiome. Indeed, a dysbiosis of the gut microbiota is associated with the pathogenesis of both intestinal and extra-intestinal disorders including inflammatory bowel disease, metabolic diseases such as obesity and diabetes mellitus type 2, and cardiovascular diseases [5]. The impact of environmental factors, including aspects of lifestyle or drug therapy on the microbiota is of major clinical interest. Diet is one of the main determinants of the microbial composition in the gut influencing diversity, distribution and abundance of microbial populations from the early stages of life [6]. Indeed, diet changes are thought to explain 57% of the total structural variation in the gut microbiota [7]. An acute change in diet has been shown to alter microbial composition within just 24 h of initiation (e.g., switching to a completely plant-based diet), with reversion to baseline within approximately 48 h of diet discontinuation [8]. According to this, there is growing interest in modifying the gut microbiota for long-term health benefits.

The microbiome analysis was part of our previously published study in which we investigated the effect of regular walnut consumption (43 g/day) on the lipid profile in healthy subjects, resulting in a significant reduction of LDL-cholesterol, apoB, triglycerides and non-HDL-cholesterol after eight weeks of intervention [9]. Evidence from recent animal and human feeding studies shows a correlation between regular nut consumption and a shift within the gut microbiome, indicating prebiotic properties of members of the tree nut family. However, the exact mechanisms by which nuts offer their prebiotic effects on microbial diversity is not fully understood [10,11]. Another issue to be addressed is, how these changes might be associated with the observed changes in lipid metabolism.

The aim of this sub study was to investigate the effect of walnut consumption on the gut microbiome composition and microbial diversity.

## 2. Materials and Methods

### 2.1. Study Design

**Figure 1:**
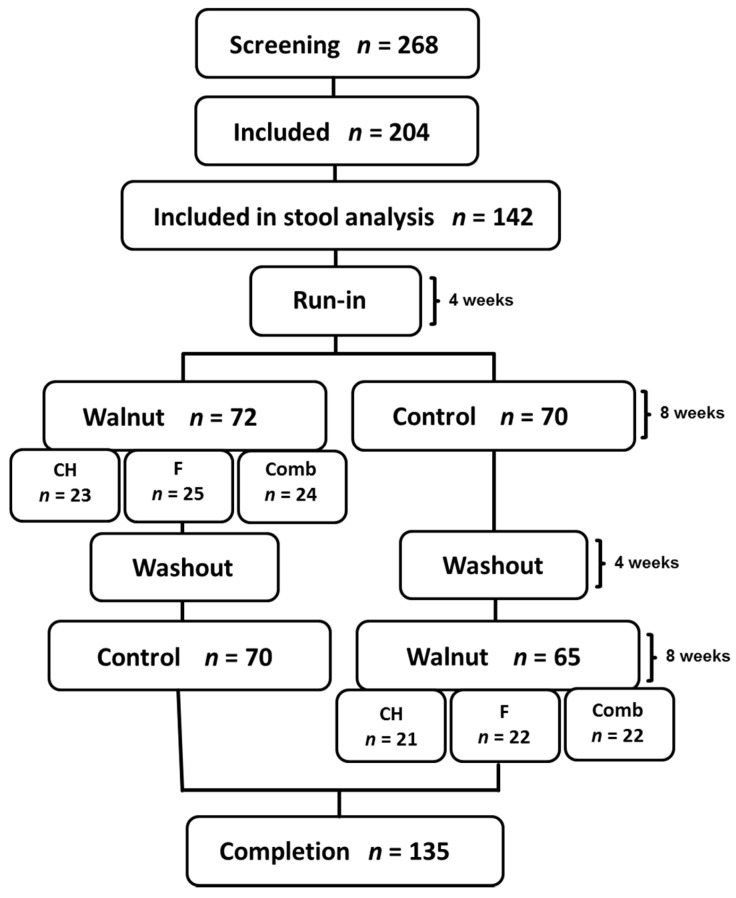
Flowchart of study subjects. In total, 204 subjects were randomized. 142 subjects were included in stool analysis. 7 subjects were excluded due to antibiotic therapy. In total, stool samples from 135 study subjects were included in statistical evaluation. CH: carbohydrate restriction; F: fat restriction; Comb: combined carbohydrate and fat restriction.

The study comprises a randomized, controlled, prospective, cross-over design as previously described [9]. Each subject followed a nut-free Western-type diet consisting of 50% carbohydrates, 35% fat (15% saturated fat), and 15% protein during a 4-week run-in period. Thereafter, subjects were randomized to 2 different diet phases, each lasting for 8 weeks (separated by a 4-week washout). One group (*n* = 96) first followed a walnut-enriched diet (43 g of shelled walnuts/day) and then switched to a nut-free control diet. The other group (*n* = 98) followed the diets in reverse order (Figure 1). Study duration was 6 months (24 weeks) for each study subject. During the walnut diet the subjects were randomized into three different diet groups, in which they were advised to reduce the intake of either carbohydrates (CH, *n* = 62; 44 with stool samples), fat (*n* = 65; 47 with stool samples), or both (*n* = 67; 46 with stool samples). They were instructed to replace either 70 g carbohydrates or 30 g of (saturated) fat with the walnuts. Subjects assigned to the third group were advised to replace both macronutrients (35 g carbohydrates and 15 g fat) with the daily walnut serving. These recommendations were food based, i.e., on the basis of individual food reports (free text), a nutritionist recommended specific measures to adjust the diet. Stool samples were collected at the end of each diet phase.

### 2.2. Study Subjects

Study participants (*n* = 204) were healthy Caucasian men and postmenopausal women older than 50 years (134 females and 60 males, age 63 ± 7 years, BMI 25.1 ± 4.0 kg/m^2^) [9]. We included healthy non-smoking subjects older than 50 years (men and postmenopausal women) with LDL-C < 190 mg/dL, triglycerides (TG) < 350 mg/dL, and a body mass index (BMI) < 35 kg/m^2^. We excluded persons with a history of cardiovascular and atherosclerotic disease, a known allergy to tree nuts, a vegan or ovo-lacto vegetarian lifestyle, and patients on regular medication (except stable treatment of thyroid disease and hypertension). Stool samples were only available in 142 of the original 204 subjects. Further 7 study subjects were subsequently excluded due to antibiotic therapy. In total, 270 stool samples (2 samples from each of the 135 study subject) were analyzed.

### 2.3. Stool Sample Collection

Subjects were instructed to collect stool samples within 24 h before the next study visit and refrigerate them until the visit. The required materials for a hygienically safe stool collection were provided by the study center. The study kit comprised disposable gloves, a sample-catching paper (Süsse Labortechnik MED AUXIL 150 × 470 mm #S1000), sample-collecting tubes including 8 mL of stool DNA stabilizer (stratec molecular #1038111100), transport bags, as well as instructions for use and a questionnaire (Bristol Stool Chart) for recording sampling conditions and sample quality [12]. Samples were immediately frozen at −20 °C, transported on dry ice and then stored at −80 °C until further analysis.

### 2.4. Sample Processing and Sequencing

The identification and comparison of microbial communities was evaluated by using high-throughput sequencing of the V3/V4 region of the 16S rRNA gene [2]. The method is based on the isolation of genomic DNA and its duplication produced by Polymerase Chain Reaction (PCR), followed by sequencing of the PCR amplicon by using a specific primer that binds to highly conserved sequences on the 16S rRNA gene [13]. Sample processing has been divided into DNA isolation, library construction by PCR, amplicon cleaning and dilution, and sequencing. DNA was isolated with a modification of the protocol by Godon et al. [14]. After isolation, DNA was purified using a silica-membrane based NucleoSpin gDNA Clean-up Kit (REF 740230.250 Machery-Nagel). The PCR was performed in duplicate and the PCR products of duplicates were pooled prior to cleaning [13]. For quality control, a selection of samples was analyzed by electrophoresis. PCR purification was performed by using AGENCOURT AMPure XP Beads (Beckman Coulter, Brea, CA, USA). The 16S rRNA gene amplicon libraries were sequenced in paired-end modus using an Illumina MiSeq.

### 2.5. Data Analysis

The resulting dataset was processed through taxonomic classification against a database of reference 16S rRNA gene sequences. After sequencing, raw data reads were assigned to their corresponding sample via demultiplexing using previously assigned barcode pairs which are unique for each sample. The demultiplexing was performed by using an in-house developed Perl script. After demultiplexing, data were analyzed using the IMNGS platform (www.imngs.org), which is based on the UPARSE approach for sequence quality check, chimera filtering, and cluster formation [15]. Output was an Operational Taxonomic Units (OTU) analysis calculating and visualizing the relative abundance of the bacterial taxa present in each sample. For quantifying alpha-diversity, the intra-sample variation is calculated. Richness gives the value of present OTUs within one sample while the diversity index estimates the number of equal species within one sample. Simpson effective counts out more weight on dominant species while Shannon is based on richness and evenness. To avoid incorrect estimation of species richness due to differential sequencing depth, only normalized counts that are above 0.5 were considered. Based on an OTU threshold of 97% similarity, UniFrac distances (a distance metric for microbial community comparison) were calculated to evaluate beta-diversity (diversity between the samples). Beta-diversity was determined by Principal Coordinate Analysis (PCoA) using both unweighted and weighted UniFrac metrices. Metric Multidimensional Scaling (MDS) and non-metric multidimensional scaling (NMDS) projections of the generalized UniFrac distances were produced and a PERMANOVA test was performed to determine statistical significant differences. Differential abundance was evaluated performing the Kruskal–Wallis rank sum test. To determine differences between groups, based on the relative abundance of occurring OTUs, a non-parametric ANOVA (Kruskal–Wallis rank sum test) was applied. Significant differences based on prevalences between groups are calculated by Fisher exact test. For downstream processing of intermediate files generated by IMNGS, a fully modular R-based pipeline (Rhea) was developed for analysis of microbial profiles [16]. Statistical significance was set at *p* ≤ 0.05. Any *p*-values less than 0.05 are shown.

### 2.6. Ethics Statement

The Study was conducted according to the guidelines in the Declaration of Helsinki and the ICH Harmonized Tripartite Guideline for Good Clinical Practice. The study protocol was approved by the ethics committee of the Faculty of Medicine of the University of Munich. After informing subjects about the study, the intervention, and side effects, all study participants provided written informed consent. The study was registered at ClinicalTrials.gov (NCT02329067) and performed between February 2015 and May 2016 at the University of Munich Medical Center. Walnuts were provided by the California Walnut Commission (Folsom, CA, USA). Sample analysis was performed at the Chair of Nutrition and Immunology (Core Facility Microbiome) of the Institute for Food and Health (ZIEL) at the Technical University of Munich (Freising, Bavaria, Germany).

## 3. Results

**Figure 2:**
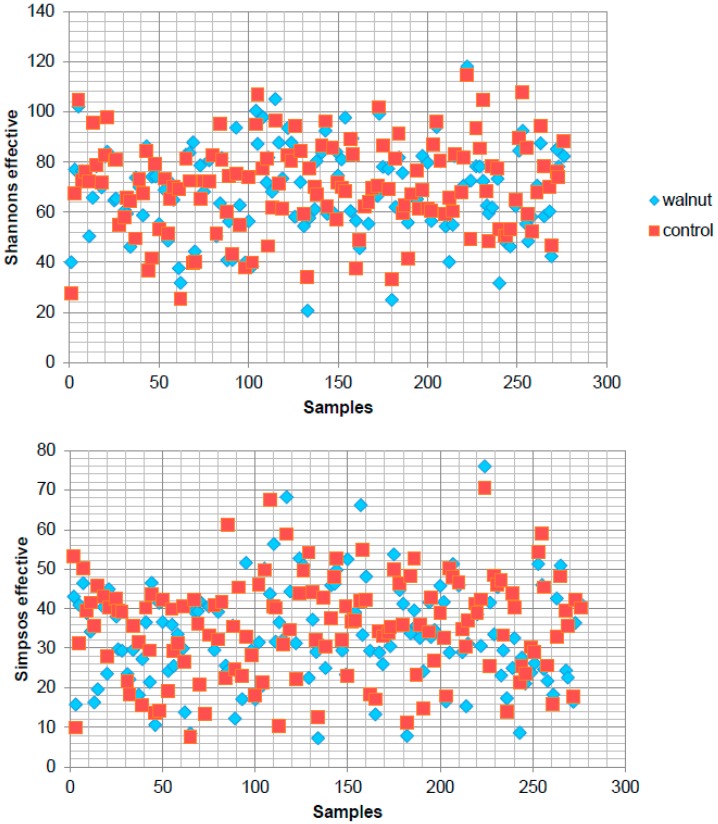
Within-sample alpha-diversity of stool of each subject collected at the end of each diet phase. Calculation of the alpha-diversity for each sample (blue: walnut diet, red: control diet) for evaluating species richness and diversity by using Shannon and Simpsons effective indices. The diversity of a microbial profile for a certain index is the number of different species related to abundance and richness.

Alpha-diversity for the walnut and control diets is shown in Figure 2. Supplementing walnuts in the diet did not significantly affect bacterial diversity measured by Shannons effective (walnut vs. control 68.189 vs. 70.118, *p* = 0.3789) and Simpsons effective (33.138 vs. 35.405, *p* = 0.0861) counts. According to this, there was no significant difference in evenness as well as in richness (179.326 vs. 179.393, *p* = 0.8522) for the walnut diet compared to the control diet.

**Figure 3:**
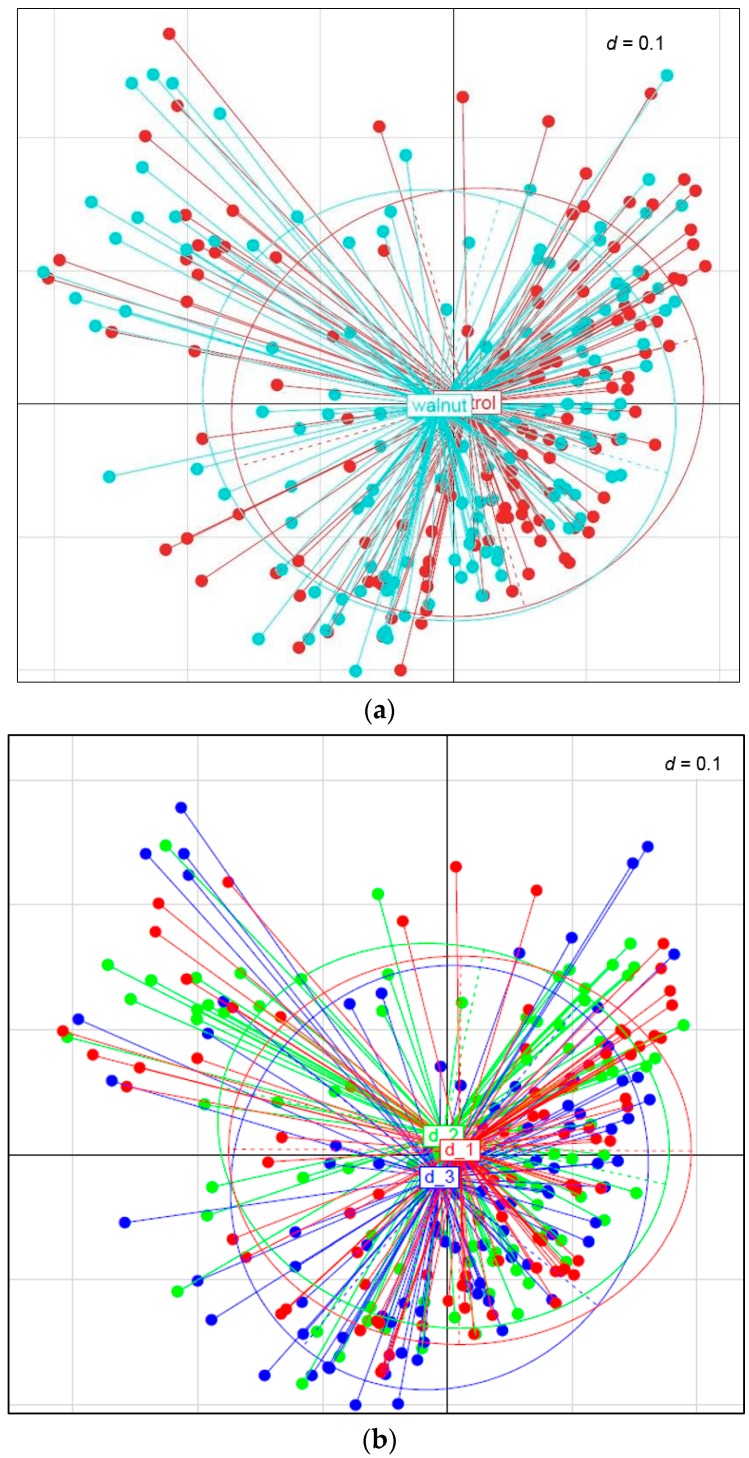
(**a**) Beta-diversity between walnut and control diet. Distinct clustering was observed between the walnut and the control diet. By using generalized UniFrac distances considering the phylogenetic distance between Operational Taxonomic Units, MDS plot indicates significant dissimilarities of approximately 5% between walnut and control (*p* = 0.02). The multidimensional distance matrix in a space of two dimensions is visualized as MDS plot. Subject’s clustering and coloring were done according to the diet type. Each dot end indicates a sample position in the microbiota dataset (blue: walnut diet, red: control diet). (**b**) Beta-diversity between three different diet types during walnut consumption. Distinct clustering was observed between the diets. By using generalized UniFrac distances considering the phylogenetic distance between Operational Taxonomic Units, MDS plot indicates significant dissimilarities of approximately 5% between the different diets (*p* = 0.026). The multidimensional distance matrix in a space of two dimensions is visualized as MDS plot. Subject’s clustering and coloring were done according to the diet type. Each dot end indicates a sample position in the microbiota dataset (red: d_1: carbohydrate restriction; green: d_2: fat restriction; blue: d_3: both).

By using generalized UniFrac distances considering the phylogenetic distance between OTUs, a multidimensional distance matrix in a space of two dimensions has been visualized by MDS and NMDS. Beta-diversity for walnut and control diet is shown as Principal Coordinate Analysis plot in Figure 3a. Generalized UniFrac analysis demonstrated a clear clustering between the walnut and the control group. MDS (metric and non-metric) indicated significant dissimilarities of approximately 5% between walnut and control (*p* = 0.02).

Generalized UniFrac analysis demonstrated a clear clustering between the different diet groups during walnut consumption (Figure 3b). Again, MDS (metric and non-metric) indicated significant dissimilarities of approximately 5% between the three diet types (*p* = 0.026).

**Figure 4:**
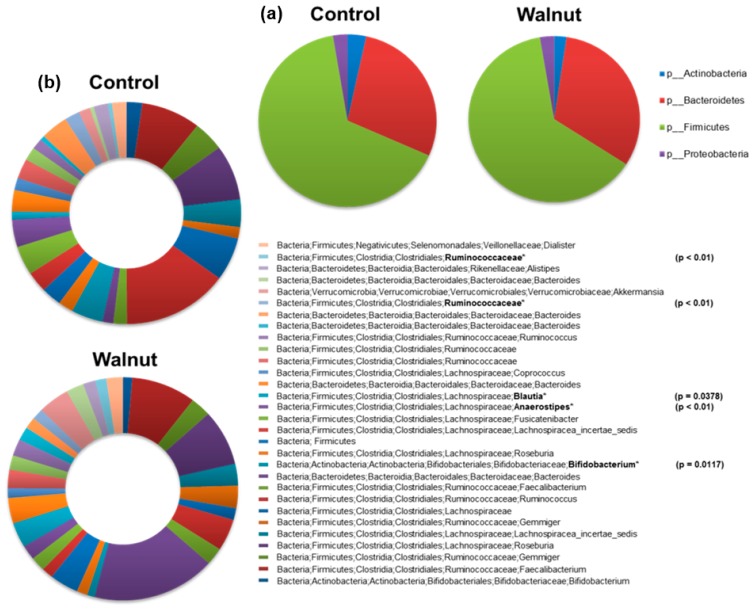
(**a**) Relative abundance of the 4 dominating bacterial phyla between the walnut and the control diet. Walnut consumption shifted the predominant phyla from *Firmicutes* (61.2% after walnut consumption vs. 63.9% after control) to *Bacteroidetes* (30.8% vs. 27.4%). Relative abundance was calculated from the relative abundance of 16S rRNA gene sequences for each bacterial community by using the IMNGS platform. (**b**) Most abundant Operational Taxonomic Units for both walnut and control phase at genus level. Significant different OTUs are marked with by using * and *p*-values. *p*-values were calculated using a pairwise Fisher test.

Although walnut consumption shifted the predominant phyla from Firmicutes (61.2% after walnut consumption vs. 63.9% after control) to Bacteroidetes (30.8% vs. 27.4% respectively), these changes in abundance were not significant. Relative abundance was calculated from the relative abundance of 16S rRNA gene sequences for each bacterial community by using the IMNGS platform. The relative changes in OTUs for the bacterial phyla are shown in Figure 4a.

The predominant bacteria at genus level (Figure 4b) were assigned to four different phyla (*Bacteroidetes*, *Firmicutes*, *Actinobacteria*, *Verrucomicrobia*), five classes (*Clostridia*, *Bacteroidia*, *Actinobacteria*, *Verrucomicrobiae*, *Negativicutes*), 5 orders (*Clostridiales*, *Bacteroidales*, *Bifidobacteriales*, *Verrucomicrobiales*, *Selenomonadales*) and seven families (*Ruminococcaceae*, *Bacteriodaceae*, *Lachnospiraceae*, *Bifidobacteriaceae*, *Veillonellaceae*, *Rikenellaceae*, *Verrucomicrobiaceae*).

**Figure 5:**
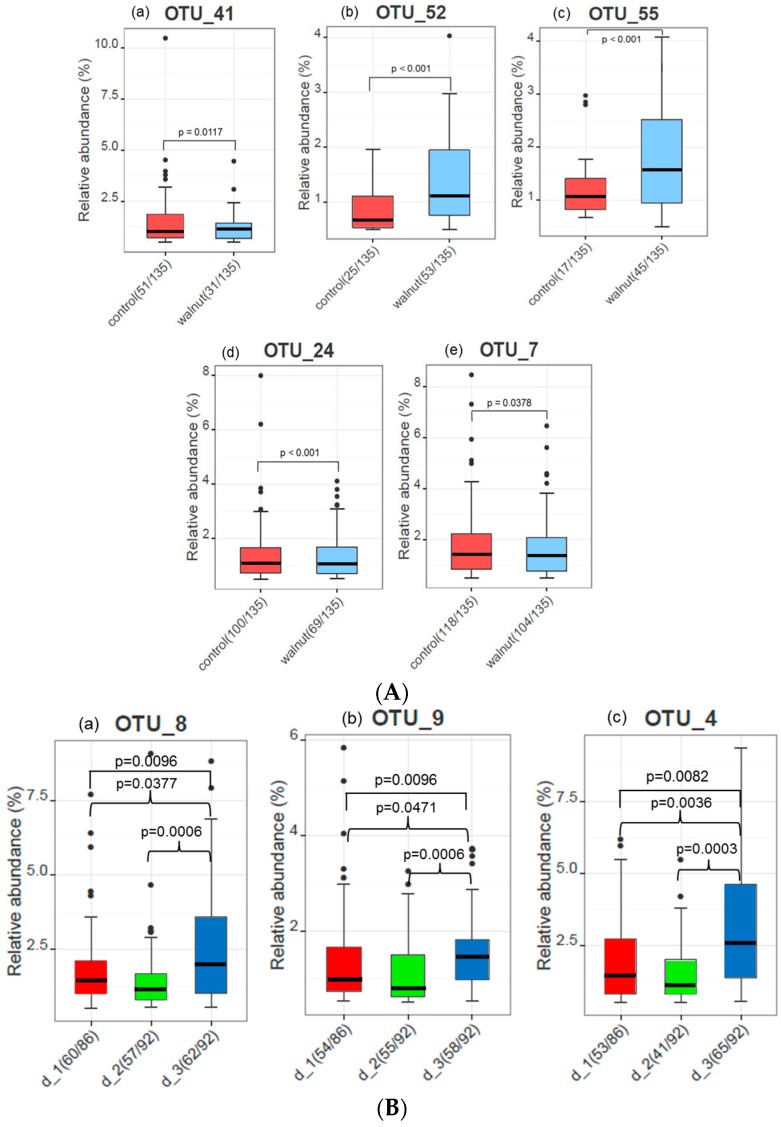
(**A**) Serial-group-comparisons between walnut and control diet. Boxplots of all significant comparisons. Since the data is not normally distributed, a non-parametric Kruskal–Wallis rank sum test and a Fisher’s exact test has been used performed in Rhea. (**a**) OTU_41: *Bacteria*; *Actinobacteria*; *Actinobacteria*; *Bifidobacteriales*; *Bifidobacteriaceae*; *Bifidobacterium*; (**b**) OTU_52: *Bacteria*; *Firmicutes*; *Clostridia*; *Clostridiales*; *Ruminococcaceae*; (**c**) OTU_55: *Bacteria; Firmicutes*; *Clostridia*; *Clostridiales*; *Ruminococcaceae*; (**d**) OTU_24: *Bacteria*; *Firmicutes*; *Clostridia*; *Clostridiales*; *Lachnospiraceae*; *Anaerostipes*; (**e**) OTU_7: *Bacteria*; *Firmicutes*; *Clostridia*; *Clostridiales*; *Lachnospiraceae*; *Blautia*. (**B**) Serial-group-comparisons between three different diets during walnut consumption. Boxplots of all significant comparisons. Since the data is not normally distributed, a non-parametric Kruskal–Wallis rank sum test and a pairwise Wilcoxon rank sum test has been used performed in Rhea. d_1: carbohydrate replacement, d_2: fat replacement, d_3: both. (**a**) OTU_8: *Bacteria*; *Firmicutes*; *Clostridia*; *Clostridiales*; *Ruminococcaceae*; *Gemmiger*; (**b**) OTU_9: *Bacteria*; *Firmicutes*; *Clostridia*; *Clostridiales*; *Lachnospiraceae*; *Fusicatenibacter*; (**c**) OTU_4: *Bacteria*; *Bacteroidetes*; *Bacteroidia*; *Bacteroidales*; *Bacteroidaceae*; *Bacteroides*.

After walnut consumption, significant shifts in the relative abundance of four members of the phyla *Firmicutes* and in one member of the phyla *Actinobacteria* could be observed (Figure 5A). A significant increase could be identified in two unknown species of the genus *Ruminococcaceae* spp. (*Clostridium* Cluster IV) (*p* < 0.02) and in the species *Bifidobacterium* of the genus *Bifidobacteriaceae* spp. (*p* < 0.02). In parallel, a significant decrease was observed in the relative abundance of two *Lachnospiraceae* species (*Clostridium* Cluster XIV) (a) *Anaerostipes* (*p* < 0.01) and (b) *Blautia* (*p* = 0.04).

Since subjects were advised to reduce either fat or carbohydrates or both during walnut consumption we also evaluated whether this affects microbiome. Comparing these three diet types during walnut consumption revealed significant shifts in the relative abundance of two members of the phyla *Firmicutes* and in one member of the phyla *Bacteroidetes* (Figure 5B). Over all groups, a significant difference could be identified in a species of the genus *Ruminococcaceae* spp. (*p* < 0.01), in one *Lachnospiraceae* species (*p* < 0.01) and in one species of the genus *Bacteroidaceae* spp. (*p* < 0.01). Pairwise testing showed significant differences between the diet types.

## 4. Discussion

Daily consumption of 43 g walnuts resulted in significant changes in composition and diversity in the gut microbiome by enhancing probiotic- and butyric acid-producing species in healthy individuals.

Obviously, diet is an important factor determining the composition of the gut microbiota. In healthy adults, bacterial clusters within the phyla *Bacteroidetes* and *Firmicutes* usually dominate the intestinal microbiota, whereas the proportions of *Actinobacteria*, *Proteobacteria* and *Verrucomicrobia* are relatively small [17]. In animal models, the ratio of *Bacteroidetes* and *Firmicutes* is altered in response to dietary changes [18]. However, although diet-induced shifts in the gut microbiota occur within a short period of time (between 1–4 days after a change in diet), these changes have been shown to be reversed just as rapidly [19,20]. Both genomic sequencings of bacterial rRNA from mice and humans indicate that a high-fat diet promotes a reduction of *Bacteroidetes*, while a fat-restricted diet results in the opposite scenario [21,22,23]. On the other hand, a high-fat Western-type diet in mice resulted in an increased abundance of *Firmicutes* and a decrease in *Bacteroidetes* [19,24,25]. In contrast, no relationship was observed between the ratio of *Bacteroidetes* and *Firmicutes* and diets low in carbohydrates [26]. Since sufficient and conclusive data from human feeding trials are missing, it is difficult to determine the mechanisms by which walnuts, as part of a Western-type diet, may confer their modulating effects on microbial distribution and changes in the ratio of the major bacterial phyla.

In our study, generalized UniFrac distances demonstrated a distinct clustering between the walnut and the control groups as well as between the different diet types, demonstrating that beta-diversity was altered by walnut consumption. MDS plotting indicated significant dissimilarities of approximately 5% between bacterial clustering during the walnut and the control diets after eight weeks of intervention (*p* = 0.02).

Overall, we identified five OTUs that were significantly associated with walnut consumption. In particular, we found an enrichment of members of the genus *Ruminococcaceae* spp. and *Bifidobacteriaceae* spp. Members of the genus *Bifidobacterium* spp. are proven to exert positive health benefits on their host due to their probiotic properties. *Bifidobacterium* spp. are the normal inhabitants of a healthy human gut, thus, a shift in their relative abundance and composition is one of the most frequent features present in various gastrointestinal diseases including inflammatory bowel disease and colorectal cancer [17,27,28,29]. *Ruminococcaceae* spp. are an abundant fraction of the human gastrointestinal microbiota and are associated with several important metabolic functions within the *Clostridiales* order (*Clostridium* sp. cluster IV) [1] due to the production of butyric acid. The short chain fatty acid butyric acid is generated from fermentation of indigestible polysaccharides [30] and provides energy for intestinal epithelial cells and contributes to host health by facilitating maintenance of colon epithelial integrity and controlling inflammatory processes [31,32]. Our findings are consistent with other studies investigating the effect of a walnut-enriched diet on the gut microbiome (consumption of 42 g/day walnuts over a period of three weeks) indicating a significant (*p* < 0.05) increase in the relative abundance within the *Clostridiales* order. [33]. Comparable results could be observed in a trial in rats showing significantly greater species diversity after ten weeks on walnut diet by increasing the abundance of probiotic-type bacteria including *Lactobacillus* spp., *Ruminococcus* spp. and *Roseburia* spp. [34].

Besides the significant increase of members of *Ruminococcaceae* spp. and *Bifidobacteriaceae* spp., our findings also showed a significant decrease of two representatives from the *Lachnospiraceae* family under walnut consumption. These butyric acid-producing microbes account for a great proportion of the *Clostridia* class (*Clostridium* spp. Cluster XIVa) and are highly abundant within the human microbiome. This contrasts with a trial evaluating the effect of walnut consumption on colon carcinogenesis in mouse models which observed an increased abundance of *Lachnospiraceae* spp. during the walnut diet [35]. However, the recommended daily serving of walnuts was higher and intervention period longer. This discrepancy must be evaluated in further trials.

While eating walnuts, subjects were instructed to either reduce fat, carbohydrates or both. In a subgroup analysis, we evaluated whether this also affects the gut microbiome. This analysis showed significant differences in the relative abundance of three microbial representatives (*Ruminococcaceae* spp., *Lachnospiraceae* sp., *Bacteroidaceae* spp.) between the different diet types, whereby no distinct tendency could be observed after pairwise comparison. Thus, it is difficult to make a clear statement about possible different effects as a consequence of macronutrient restriction. As previously mentioned, our subjects did not fully comply with the recommended diet (i.e., substitution of carbohydrates or fat or both for walnuts), indicating that subjects had a similar diet, despite different recommendations [9]. Since our study was designed as “free-living-study” it has to be kept in mind that there are probable discrepancies in the intake of further phytonutrients (including flavonoids, carotenoids, polyphenols, etc.) and dietary fiber intake, which may also induce changes in the gut microbiome (although we did not observe any change in overall fiber intake). However, the study did not focus on changes in these components, particularly since the study relied on self-reported food records making it difficult to correctly estimate phytonutrient intake. The effect of these components can only be addressed by a different study design.

The exact mechanisms by which walnuts may exert their beneficial health effects have not yet been sufficiently investigated. The short chain fatty acid butyrate may beneficially affect metabolic and inflammatory processes and, thus, obesity, diabetes and inflammatory bowel diseases [36,37]. However, only few feeding trials have examined the prebiotic effect of nuts, especially walnuts. Thus, the exact mechanism by which they shift the relative abundance of microbial communities and modulate fluctuations in the microflora composition in the gut in favor of butyrate-producing microbes is unknown. Furthermore, non-digestible material from nuts, mainly polyphenols and polysaccharides including dietary fiber seem to have a prebiotic effect by increasing *Lactobacillus* spp. and *Bifidobacterium* spp. growth and fermentation of indigestible components to short-chain fatty acids including butyrate [10,38,39] that may also alter activity of intestinal microbial enzymes [40].

In contrast to a higher beta-diversity, the change in alpha-diversity was not significantly different between the walnut and the control groups. This indicates that, under walnut consumption, the gut microbiota showed a slightly lower diversity than under the control diet; however, this difference was not significant. Walnut consumption shifted (not significantly) the predominant phyla from *Firmicutes* (61.2% after walnut consumption vs. 63.9% after control) to *Bacteroidetes* (30.8% vs. 27.4%) (Figure 4). As mentioned above, the correlation between specific diets and shifts within the *Firmicutes*/*Bacteroidetes* ratio is a matter of controversy [21,22,23,24,25,26]. Our data do not agree with the results of two previous animal feeding studies demonstrating that walnuts significantly altered the relative abundance of these two major gut bacterial phyla independent of the length of walnut consumption [34,41]. However, study conditions are hardly comparable due to the different walnut serving sizes and varying study durations. To date, no human feeding trials are available to discuss the effect of walnut consumption on the *Firmicutes*/*Bacteroidetes* ratio.

As a first conclusion, our data indicate a correlation between walnut consumption and a shift within the gut microbiome, suggesting that a regular supplementation might offer prebiotic and probiotic benefits by improving the microbiome composition and diversity.

Recently, three papers described the prebiotic properties of other members of the tree nut family in human clinical feeding trials. One study determined the effects of almond and pistachio consumption on gut microbiota composition in humans. The effect of pistachios was much stronger than that of almonds and resulted in an increase in potentially beneficial butyrate-producing bacteria in the phylum *Firmicutes* [42]. Comparable results have been demonstrated by another human feeding trial with a similar initial hypothesis but over a much shorter intervention period of only 18 days. The effect of pistachio consumption on gut microbiota composition was again much stronger compared to that of almond consumption. It was concluded that almonds and especially pistachios can affect the composition of the fecal bacterial microbiota [43]. In vitro and in vivo studies analyzed the prebiotic effect and fermentation properties of raw and roasted almonds, as well as almond seed and almond skin [40,44,45,46,47]. Both raw and roasted almonds showed potential prebiotic effects on intestinal bacteria and metabolic activities, showing a stimulatory effect on fecal *Lactobacillus* spp., and *Bifidobacterium* spp. [40]. Significant increases in the abundance of *Bifidobacterium* spp. and *Lactobacillus* spp. could also be observed in fecal samples as a consequence of both raw almond and almond skin supplementation [47].

Although our findings are only observational, the results indicate that nuts (especially walnuts) may represent an important dietary supplement not only to positively influence blood lipids but also to improve gut microbiome health. It is unclear if and how the changes in the microbiome are linked to the observed changes in fasting lipid metabolism [9]. The study design and the high variability in the observed changes in the microbiome preclude any valid conclusions at this point. Interestingly, there are only very few studies investigating the effect of statins on gut bacteria. It has been hypothesized that gut bacteria may cause inherent differences in the way subjects metabolize and benefit from therapeutic agents due to higher levels of bacterial-derived bile acids [48]. Furthermore, gut microbiota analysis in mice treated with hypolipidemic drugs revealed a modification in composition in favor of probiotic-type bacteria from *Lactobacillus* spp. [49]. However, the exact mechanisms by which cholesterol-lowering substances may interact with the human gut microbiome have not been sufficiently investigated. Another interesting aspect that should be considered for further investigations is the finding that certain metabolites strongly correlate with microbial community structures which would allow gaining insights into microbiome–host interactions, also in context of certain diseases and therapeutic interventions [50,51]. The analysis of metabolic fingerprints might be useful to understand how microbial structures are influenced by regular walnut consumption.

More interventional nutritional studies might be required to quantify the underlying mechanisms by which walnut components influence microbiome composition and how the abundance of butyrate-producing bacteria is increased. Furthermore, further evaluation regarding whether these observed changes are preserved during longer walnut consumption is required.

## 5. Conclusions

Current study results show a shift within the composition of microbial communities in the human gut under nut consumption. This shift is characterized by an increase in the relative abundance of potentially beneficial butyrate-producing bacteria. In our study, we showed that daily intake of 43 g walnuts over eight weeks significantly affected the gut microbiome by enhancing probiotic- and butyric acid-producing bacteria in healthy individuals. It is unclear whether these changes are preserved during longer walnut supplementation and how these changes are associated with the observed changes in lipid metabolism. More human intervention trials investigating different servings of nuts over a longer time period might be useful to further evaluate the prebiotic properties of walnut consumption.

## References

[1] Rajilic-Stojanovic M., de Vos W.M. (2014). The first 1000 cultured species of the human gastrointestinal microbiota. FEMS Microbiol. Rev..

[2] Woese C.R., Fox G.E. (1977). Phylogenetic structure of the prokaryotic domain: The primary kingdoms. Proc. Natl. Acad. Sci. USA.

[3] Singh R.K., Chang H.W., Yan D., Lee K.M., Ucmak D., Wong K., Abrouk M., Farahnik B., Nakamura M., Zhu T.H. (2017). Influence of diet on the gut microbiome and implications for human health. J. Transl. Med..

[4] Manco M., Putignani L., Bottazzo G.F. (2010). Gut microbiota, lipopolysaccharides, and innate immunity in the pathogenesis of obesity and cardiovascular risk. Endocr. Rev..

[5] Carding S., Verbeke K., Vipond D.T., Corfe B.M., Owen L.J. (2015). Dysbiosis of the gut microbiota in disease. Microb. Ecol. Health Dis..

[6] Del Chierico F., Vernocchi P., Dallapiccola B., Putignani L. (2014). Mediterranean diet and health: Food effects on gut microbiota and disease control. Int. J. Mol. Sci..

[7] Zhang C., Zhang M., Wang S., Han R., Cao Y., Hua W., Mao Y., Zhang X., Pang X., Wei C. (2010). Interactions between gut microbiota, host genetics and diet relevant to development of metabolic syndromes in mice. ISME J..

[8] David L.A., Maurice C.F., Carmody R.N., Gootenberg D.B., Button J.E., Wolfe B.E., Ling A.V., Devlin A.S., Varma Y., Fischbach M.A. (2014). Diet rapidly and reproducibly alters the human gut microbiome. Nature.

[9] Bamberger C., Rossmeier A., Lechner K., Wu L., Waldmann E., Stark R.G., Altenhofer J., Henze K., Parhofer K.G. (2017). A walnut-enriched diet reduces lipids in healthy Caucasian subjects, independent of recommended macronutrient replacement and time point of consumption: A prospective, randomized, controlled trial. Nutrients.

[10] Lamuel-Raventos R.M., Onge M.S. (2017). Prebiotic nut compounds and human microbiota. Crit. Rev. Food Sci. Nutr..

[11] Burns A.M., Zitt M.A., Rowe C.C., Langkamp-Henken B., Mai V., Nieves C., Ukhanova M., Christman M.C., Dahl W.J. (2016). Diet quality improves for parents and children when almonds are incorporated into their daily diet: A randomized, crossover study. Nutr. Res..

[12] Lewis S.J., Heaton K.W. (1997). Stool form scale as a useful guide to intestinal transit time. Scand. J. Gastroenterol..

[13] Klindworth A., Pruesse E., Schweer T., Peplies J., Quast C., Horn M., Glockner F.O. (2013). Evaluation of general 16S ribosomal RNA gene PCR primers for classical and next-generation sequencing-based diversity studies. Nucleic Acids Res..

[14] Godon J.J., Zumstein E., Dabert P., Habouzit F., Moletta R. (1997). Molecular microbial diversity of an anaerobic digestor as determined by small-subunit rDNA sequence analysis. Appl. Environ. Microbiol..

[15] Lagkouvardos I., Joseph D., Kapfhammer M., Giritli S., Horn M., Haller D., Clavel T. (2016). IMNGS: A comprehensive open resource of processed 16S rRNA microbial profiles for ecology and diversity studies. Sci. Rep..

[16] Lagkouvardos I., Fischer S., Kumar N., Clavel T. (2017). Rhea: A transparent and modular R pipeline for microbial profiling based on 16S rRNA gene amplicons. PeerJ.

[17] Tojo R., Suarez A., Clemente M.G., de los Reyes-Gavilan C.G., Margolles A., Gueimonde M., Ruas-Madiedo P. (2014). Intestinal microbiota in health and disease: Role of bifidobacteria in gut homeostasis. World J. Gastroenterol..

[18] Eckburg P.B., Bik E.M., Bernstein C.N., Purdom E., Dethlefsen L., Sargent M., Gill S.R., Nelson K.E., Relman D.A. (2005). Diversity of the human intestinal microbial flora. Science.

[19] Turnbaugh P.J., Backhed F., Fulton L., Gordon J.I. (2008). Diet-induced obesity is linked to marked but reversible alterations in the mouse distal gut microbiome. Cell Host Microbe.

[20] Walker A.W., Ince J., Duncan S.H., Webster L.M., Holtrop G., Ze X., Brown D., Stares M.D., Scott P., Bergerat A. (2011). Dominant and diet-responsive groups of bacteria within the human colonic microbiota. ISME J..

[21] Wolf K.J., Lorenz R.G. (2012). Gut microbiota and obesity. Curr. Obes. Rep..

[22] Tilg H., Kaser A. (2011). Gut microbiome, obesity, and metabolic dysfunction. J. Clin. Investig..

[23] Ley R.E., Turnbaugh P.J., Klein S., Gordon J.I. (2006). Microbial ecology: Human gut microbes associated with obesity. Nature.

[24] Turnbaugh P.J., Ridaura V.K., Faith J.J., Rey F.E., Knight R., Gordon J.I. (2009). The effect of diet on the human gut microbiome: A metagenomic analysis in humanized gnotobiotic mice. Sci. Transl. Med..

[25] Hildebrandt M.A., Hoffmann C., Sherrill-Mix S.A., Keilbaugh S.A., Hamady M., Chen Y.Y., Knight R., Ahima R.S., Bushman F., Wu G.D. (2009). High-fat diet determines the composition of the murine gut microbiome independently of obesity. Gastroenterology.

[26] Duncan S.H., Belenguer A., Holtrop G., Johnstone A.M., Flint H.J., Lobley G.E. (2007). Reduced dietary intake of carbohydrates by obese subjects results in decreased concentrations of butyrate and butyrate-producing bacteria in feces. Appl. Environ. Microbiol..

[27] O’Callaghan A., van Sinderen D. (2016). Bifidobacteria and their role as members of the human gut microbiota. Front. Microbiol..

[28] Riviere A., Selak M., Lantin D., Leroy F., De Vuyst L. (2016). Bifidobacteria and butyrate-producing colon bacteria: Importance and strategies for their stimulation in the human gut. Front. Microbiol..

[29] Druart C., Alligier M., Salazar N., Neyrinck A.M., Delzenne N.M. (2014). Modulation of the gut microbiota by nutrients with prebiotic and probiotic properties. Adv. Nutr..

[30] Venkataraman A., Sieber J.R., Schmidt A.W., Waldron C., Theis K.R., Schmidt T.M. (2016). Variable responses of human microbiomes to dietary supplementation with resistant starch. Microbiome.

[31] Morrison D.J., Preston T. (2016). Formation of short chain fatty acids by the gut microbiota and their impact on human metabolism. Gut Microbes.

[32] Lopetuso L.R., Scaldaferri F., Petito V., Gasbarrini A. (2013). Commensal clostridia: Leading players in the maintenance of gut homeostasis. Gut Pathog..

[33] Guetterman H.M., Swanson K.S., Novotny J.A., Baer D.J., Holscher H.D. (2016). Walnut consumption influences the human gut microbiome. FASEB J..

[34] Byerley L.O., Samuelson D., Blanchard E.t., Luo M., Lorenzen B.N., Banks S., Ponder M.A., Welsh D.A., Taylor C.M. (2017). Changes in the gut microbial communities following addition of walnuts to the diet. J. Nutr. Biochem..

[35] Nakanishi M., Chen Y., Qendro V., Miyamoto S., Weinstock E., Weinstock G.M., Rosenberg D.W. (2016). Effects of walnut consumption on colon carcinogenesis and microbial community structure. Cancer Prev. Res. (Phila.).

[36] Brahe L.K., Astrup A., Larsen L.H. (2013). Is butyrate the link between diet, intestinal microbiota and obesity-related metabolic diseases?. Obes. Rev..

[37] Puertollano E., Kolida S., Yaqoob P. (2014). Biological significance of short-chain fatty acid metabolism by the intestinal microbiome. Curr. Opin. Clin. Nutr. Metab. Care.

[38] Selma M.V., Espin J.C., Tomas-Barberan F.A. (2009). Interaction between phenolics and gut microbiota: Role in human health. J. Agric. Food Chem..

[39] Cardona F., Andres-Lacueva C., Tulipani S., Tinahones F.J., Queipo-Ortuno M.I. (2013). Benefits of polyphenols on gut microbiota and implications in human health. J. Nutr. Biochem..

[40] Liu Z., Wang W., Huang G., Zhang W., Ni L. (2016). In vitro and in vivo evaluation of the prebiotic effect of raw and roasted almonds (*Prunus amygdalus*). J. Sci. Food Agric..

[41] Byerley L., Ponder M., Lorenzo B., Banks S., Taylor C., Luo M., Blanchard E., Welsh D. (2015). Walnut consumption changes the relative abundance of bacteroidetes and firmicutes in the gut. FASEB J..

[42] Mai V., Fredborg M., Ukhanova M., Wang X., Daniel S., Novotny J., Gebauer S., Baer D. (2012). Human gut microbiota changes after consumption of almonds or pistachios. FASEB J..

[43] Ukhanova M., Wang X., Baer D.J., Novotny J.A., Fredborg M., Mai V. (2014). Effects of almond and pistachio consumption on gut microbiota composition in a randomised cross-over human feeding study. Br. J. Nutr..

[44] Mandalari G., Faulks R.M., Bisignano C., Waldron K.W., Narbad A., Wickham M.S. (2010). In vitro evaluation of the prebiotic properties of almond skins (*Amygdalus communis* L.). FEMS Microbiol. Lett..

[45] Mandalari G., Nueno-Palop C., Bisignano G., Wickham M.S., Narbad A. (2008). Potential prebiotic properties of almond (*Amygdalus communis* L.) seeds. Appl. Environ. Microbiol..

[46] Ellis P.R., Kendall C.W., Ren Y., Parker C., Pacy J.F., Waldron K.W., Jenkins D.J. (2004). Role of cell walls in the bioaccessibility of lipids in almond seeds. Am. J. Clin. Nutr..

[47] Liu Z., Lin X., Huang G., Zhang W., Rao P., Ni L. (2014). Prebiotic effects of almonds and almond skins on intestinal microbiota in healthy adult humans. Anaerobe.

[48] Kaddurah-Daouk R., Baillie R.A., Zhu H., Zeng Z.B., Wiest M.M., Nguyen U.T., Wojnoonski K., Watkins S.M., Trupp M., Krauss R.M. (2011). Enteric microbiome metabolites correlate with response to simvastatin treatment. PLoS ONE.

[49] Catry E., Pachikian B.D., Salazar N., Neyrinck A.M., Cani P.D., Delzenne N.M. (2015). Ezetimibe and simvastatin modulate gut microbiota and expression of genes related to cholesterol metabolism. Life Sci..

[50] McHardy I.H., Goudarzi M., Tong M., Ruegger P.M., Schwager E., Weger J.R., Graeber T.G., Sonnenburg J.L., Horvath S., Huttenhower C. (2013). Integrative analysis of the microbiome and metabolome of the human intestinal mucosal surface reveals exquisite inter-relationships. Microbiome.

[51] Daliri E.B., Wei S., Oh D.H., Lee B.H. (2017). The human microbiome and metabolomics: Current concepts and applications. Crit. Rev. Food Sci. Nutr..

